# HCMV latency: what regulates the regulators?

**DOI:** 10.1007/s00430-019-00581-1

**Published:** 2019-02-14

**Authors:** Elizabeth Elder, John Sinclair

**Affiliations:** Department of Medicine, University of Cambridge, Box 157 Addenbrooke’s Hospital, Hills Road, Cambridge, CB2 0QQ UK

**Keywords:** Cytomegalovirus, Latency, Chromatin, US28, Viral reservoir, Cell signalling

## Abstract

Human cytomegalovirus (HCMV) latency and reactivation is regulated by the chromatin structure at the major immediate early promoter (MIEP) within myeloid cells. Both cellular and viral factors are known to control this promoter during latency, here we will review the known mechanisms for MIEP regulation during latency. We will then focus on the virally encoded G-protein coupled receptor, US28, which suppresses the MIEP in early myeloid lineage cells. The importance of this function is underlined by the fact that US28 is essential for HCMV latency in CD34^+^ progenitor cells and CD14^+^ monocytes. We will describe cellular signalling pathways modulated by US28 to direct MIEP suppression during latency and demonstrate how US28 is able to ‘regulate the regulators’ of HCMV latency. Finally, we will describe how cell-surface US28 can be a target for antiviral therapies directed at the latent viral reservoir.

## Introduction

Human cytomegalovirus (HCMV) persists for the lifetime of the host, a process underpinned by the establishment of latency in specific cell types. Sporadic reactivation of HCMV is thought to be well-controlled by host immune responses resulting in subclinical events, but HCMV reactivation poses a grave risk to immunocompromised individuals, especially immunosuppressed organ transplant recipients. All current therapies for HCMV disease target the lytic phase of infection, and therefore cannot reduce or remove latent reservoirs in either the donor organ or recipient. Here, we discuss our molecular understanding of latency and reactivation and how our insights have yielded novel ways to target the latent reservoir.

## HCMV latency and reactivation is regulated by chromatin structure at the major immediate early promoter

Latent carriage of HCMV requires the maintenance of the viral genome in the absence of the production of infectious virus particles; however, under certain conditions, virus is able to reactivate and produce new virus particles. This ability to reactivate sets latency apart from abortive infection and cellular differentiation is intimately linked with both latency and reactivation.

One important site of human cytomegalovirus latency is in cells of the early myeloid lineage. CD34^+^ progenitors and their derivatives, including granulocyte–macrophage progenitors and CD14^+^ monocytes, are latently infected in seropositive individuals [1–4]. Reactivation of HCMV has been observed in vitro and ex vivo upon differentiation of CD34^+^ progenitor cells into mature dendritic cells or macrophages [5–8]. While differentiation-independent virus reactivation has been recently reported in an immortal myeloid cell line [9], the mechanism of reactivation from latency has only been extensively described during myeloid cell differentiation.

A key hallmark of latency is the suppression of immediate early (IE) gene expression, and conversely, the earliest events in reactivation are the activation of IE gene expression. The absence of IE1/IE72 and IE2/IE86 transcripts during latency is a common theme throughout the results of multiple analyses of viral gene expression in latently infected cells, both ex vivo and in in vitro models [3, 4, 7, 10–12]. It follows that control of IE gene expression is an important determinant of latency and reactivation. IE gene expression is regulated by the major immediate early promoter and enhancer regions, which will be referred to, here, simply as the MIEP. Encompassing over 1 kb of DNA, regions within the MIEP can be bound by activatory or repressive transcription factors, and since HCMV DNA is rapidly chromatinised upon entry into the nucleus, the MIEP is subject to regulation by chromatin structure [13–17].

**Fig. 1:**
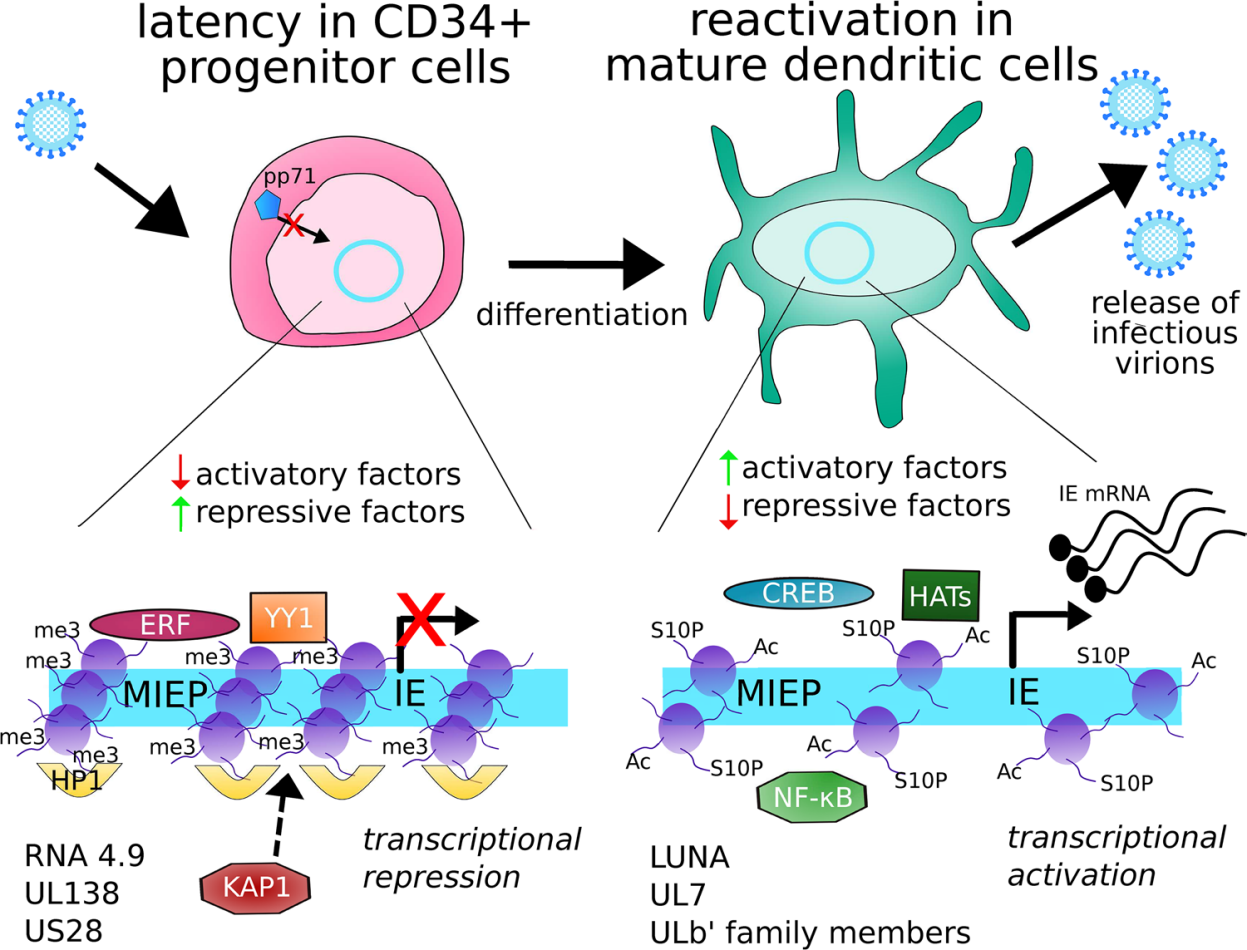
Regulation of HCMV latency and reactivation during myeloid differentiation. HCMV infects CD34^+^ progenitor cells and establishes latency (top left). The HCMV genome is maintained in the nucleus as an episome (blue circle) and is chromatinised. The MIEP (represented bottom left) is prevented from driving IE gene expression by a repressive chromatin state. Histones (purple) are trimethylated (me3) at H3K9 and H3K27. The repressive factor HP1 associates with the MIEP, as do ERF and YY1, and KAP1 acts to suppress the MIEP from distal binding sites. Latency-associated viral factors (listed) contribute to MIEP suppression, and the activatory factor pp71 is excluded from the nucleus. During differentiation-induced reactivation in mature dendritic cells or macrophages (top right), transcription of IE genes is activated leading to full lytic replication and release of infectious virions. As a result of differentiation, the chromatin structure around the MIEP is more open (bottom right), and activatory histone marks including histone acetylation (Ac) and H3-serine-10-phosphorylation (S10P) are present. Activated CREB and NF-κB become associated with the MIEP, as do histone acetyl transferases (HATs). Several viral factors are reported to be important for reactivation in myeloid cells, including LUNA, UL7, and certain members of the ULb’ family

Analyses of chromatin structure at the major immediate early promoter reveals that latency coincides with a repressive chromatin structure around the MIEP, including the presence of the heterochromatin marker HP1 [7, 8, 18], as well as the histone modifications histone-H3-lysine-27-trimethylation (H3K27me3) and histone-H3-lysine-9-trimethylation (H3K9me3) [19, 20] (see also Fig. 1). Histone deacetylase (HDAC) activity is also important for maintaining a repressed chromatin state; treatment of latently infected monocytes with HDAC inhibitors leads to transient activation of IE gene expression [21].

The differentiation of CD34^+^ progenitor cells, which can carry latent HCMV in vivo, into mature dendritic cells results in the removal of repressive H3K27me3 and H3K9me3 marks and associated HP1 from the MIEP [7, 8, 19, 20]. Additionally, phosphorylation of histone H3-serine-10 (H3S10P) at the MIEP has been shown to precede the removal of repressive marks during the differentiation of experimentally infected monocytes into immature dendritic cells [22]. Acetylation of histone H4 has also been demonstrated during reactivation from latency in maturing dendritic cells [7, 8]. As such, an open chromatin structure around the MIEP permits the initiation of IE transcription which is necessary for reactivation.

## Cellular and viral factors control the MIEP during latency

Clearly, a repressive chromatin structure around the MIEP must be established during latency in myeloid progenitors and then modified during reactivation to permit efficient IE gene expression in differentiated dendritic cells and macrophages (Fig. 1). We know that this process relies upon both cellular and viral factors; these can function by direct binding to the MIEP or by indirect mechanisms and have either activatory or repressive functions. A long-standing hypothesis states that it is the balance of these activatory or repressive factors that then controls whether or not the MIEP drives IE gene transcription, and that cellular differentiation must alter this balance [23–25].

Some host cell transcription factors bind directly to the overlapping 18, 19, and 21 bp repeats within the MIEP as well as other motifs in more upstream sequences (direct acting factors) [17]. This includes the repressive factors YY1 and ERF, and the activatory factors NF-κB and CREB, which have been discussed in the context of latency and reactivation previously [17]. In brief, in undifferentiated, non-permissive cells, the repressive factors YY1 [26] and ERF [27, 28] bind to the 21 bp repeats. ERF is thought to recruit HDAC1 to the MIEP, thus providing a link between transcription factor binding to specific DNA sequence motifs and epigenetic modification. Interestingly, absolute levels of YY1 decreased during differentiation of the non-permissive NT2 cell line [26].

KAP1 was more recently identified as a chromatin organiser that can mediate repression during latency [20]. While not strictly a DNA-binding protein, KAP1 was found to associate with a number of sites on the HCMV genome in CD34^+^ progenitor cells, and KAP1 deposition at these sites correlated with the presence of the KAP1 effector SETDB1, as well as HP1 and H3K9me marks at the MIEP. When KAP1 was depleted, these marks were lost and the virus entered lytic replication in the absence of cellular differentiation. Furthermore, KAP1 activity was shown to be repressed during lytic infection by mTOR-mediated phosphorylation, thus providing a potential mechanism for exiting latency.

Other host factors which do not, themselves, bind to viral DNA are thought to control the presence or activation of other direct-acting factors. As discussed, mTOR-mediated phosphorylation of KAP1 abrogates the repressive activity of KAP1, implying that mTOR is important for regulating latency. Other host kinases are also important. Linking reactivation with cellular differentiation, IL-6/LPS-stimulated activation of ERK-MAPK pathways was shown to be crucial for inducing MIEP activity in maturing dendritic cells [22, 29]. CREB is phosphorylated by the downstream kinase MSK, which is required for its activation at the MIEP. The absence of this signalling during latency in myeloid progenitors may, therefore, prevent CREB activity.

The role of viral factors during latency is becoming more appreciated (Fig. 1) [30]. Viral gene expression during experimental and natural latency, as measured by RNAseq, has recently been found to be rather broad and complex [19, 31, 32] than when compared with earlier microarray or targeted RT-PCR studies of latently infected cells [10, 33–35]. In addition, it is important to consider the numerous viral factors that may enter myeloid cells as components of the virion. For example, the viral long non-coding RNA 4.9 has been reported to bind the MIEP and recruit the repressor complex PRC2 to the MIEP [19]. The viral transactivator pp71 is excluded from the nucleus of non-permissive cells, and since pp71 has been shown to be important for antagonising the functions of PML bodies during lytic infection, exclusion of pp71 may help mediate PML-mediated repression of the MIEP [36]. However, other reports in different systems note that knockdown of PML components had no effect on the establishment of latency [37, 38] and, furthermore, a recent study found that the viral factor LUNA actually disperses PML bodies during latent infection in CD34^+^ cells [39].

The latency-associated gene product UL138 does not localise to the nucleus but instead manipulates cellular signalling pathways from the ER, probably in concert and in opposition with other members of the ULb’ region [40]. In brief, UL138 has been reported to repress MIEP activity, in part by blocking histone lysine-demethylase activity during latency [41] and also likely via manipulation of EGFR signalling [41, 42]. Meanwhile, other viral factors promote reactivation from latency, including LUNA and UL7 [39, 43, 44].

The virally-encoded G-protein coupled receptor US28 is expressed during lytic and latent infections, as well as coming in with the virion [45] and has recently gained prominence as an essential protein for latency. In the remainder of this review, we will discuss how US28 is able to alter cell signalling in a differentiation dependent manner, and thus promote latency in myeloid progenitor cells.

## US28 is essential for HCMV latency in CD34^+^ progenitor cells and CD14^+^ monocytes

It has been known for some time that US28 is expressed during latent infection of myeloid cells [45–49] but the functions of US28 have mostly been described for lytic infection. During the replication cycle of HCMV, US28 acts as a chemokine receptor homologue, binding CXXXC and CC chemokines [50, 51], but US28 can also signal constitutively [52, 53]. A comprehensive summary of US28 signalling functions during lytic infection, including cell type specificity, ligand interactions, and G-protein usage, was recently published [54].

However, intriguingly, US28 gene deletion viruses (ΔUS28) fail to establish latency in CD34^+^ progenitor cells [45, 49] and CD14^+^ monocytes [55], instead they fail to repress the MIEP, driving IE expression and full lytic cycle with the eventual release of infectious viral particles. Removing US28 uncouples permissiveness from cellular differentiation, since monocytes infected with ΔUS28 HCMV undergo lytic infection but do not show differentiation-specific cell surface markers [55]. US28 was shown to be expressed and translated de novo as well as entering the cell with the virion [45] and it has now become clear that both incoming US28 and de novo expressed US28 are important for the establishment and ongoing maintenance of latency in myeloid progenitor cells [56]. Other sites of HCMV latency or low level persistence, such as neuronal cells and endothelial cells, have been suggested but, as yet, these have not been confirmed in vivo and there is no evidence that US28 is required to negatively regulate the MIEP during latent/persistent infections of these cell types in vitro [54].

Use of characterised mutants of US28 has elucidated some US28-mediated functions that are important for latency. The Y16F mutant removes some ligand binding activity [57] and the R129A mutant abrogates coupling of G-proteins to the DRY box motif of US28 [58–61]. Expression of US28-WT in trans rescues latency-establishment in cell line models with the ΔUS28 virus. Similarly, expression of US28-Y16F in trans could also complement the US28 deletion virus suggesting that certain modes of ligand binding may not be necessary for the latency-associated function of US28 [55]. However, deletion of the entire ligand binding domain of US28 in the virus causes lytic infection in myeloid cells [56], which is perhaps explained by recent work demonstrating the multiple modes by which US28 can bind a wide array of ligands [62]. It is clear, however, that either expression of US28-R129A in trans or within the virus, fails to lead to latency establishment, providing clear evidence that US28-signalling via G proteins is essential for latency [55, 56]. The way that US28 signalling manipulates the host environment to support latency is therefore of great interest and under intense study.

## US28 alters cellular signalling within myeloid cells

**Fig. 2:**
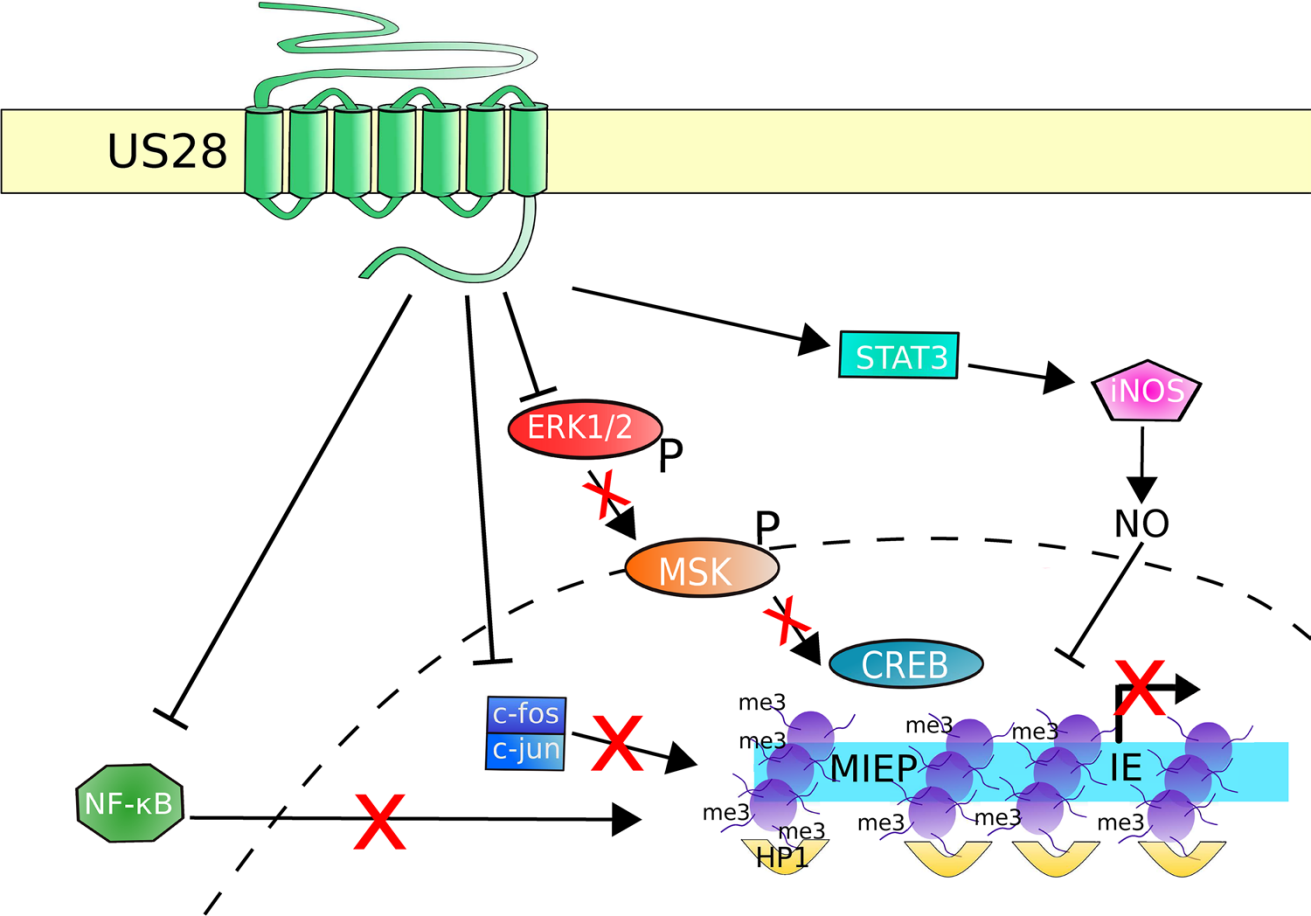
US28 controls several signaling pathways to suppress the MIEP in early myeloid lineage cells. US28 is present at the cell surface, and probably other membranes, of latently infected cells. Here, it attenuates several signaling pathways and transcription factors, including NF-κB, c-fos, and ERK1/2. NF-κB can no longer enter the nucleus (dashed line), nor bind and activate the MIEP. c-fos typically forms a dimer with c-jun to form the AP1 complex; US28 causes loss of c-fos, the AP1 complex does not form and thus cannot activate the MIEP. Attenuation of ERK1/2 causes loss of ERK1/2 phosphorylation (P) and subsequent activation of MSK and, therefore, MSK does not phosphorylate and activate CREB. Inactive CREB cannot activate the MIEP. US28 is also reported to activate the STAT3-iNOS signaling axis, leading to nitric oxide (NO) production. NO suppresses the MIEP in myeloid cells by unknown mechanisms. By these, and probably other pathways, US28 helps establish and maintain a repressive chromatin structure at the MIEP, and a lack of IE gene expression

Analysis of the activation states of cellular kinases during latency with US28 expressed in isolation in myeloid cells has revealed several signalling pathways that are important for latency (summarised in Fig. 2). Infection of CD34^+^ progenitor cells with WT virus, but not ΔUS28 HCMV, drives activation of the STAT3-iNOS pathway, and the resultant nitric oxide production was shown to suppress the MIEP [49]. Furthermore, these authors showed that presence of US28 in the context of latent infection may reprogramme infected cells to become immunosuppressive monocytes akin to myeloid-derived suppressor cells, rather than conventional monocytes or, indeed, parts of other myeloid or lymphoid lineages.

Additionally, US28 has been found to attenuate several cellular signalling pathways, such as ERK, MSK, NF-κB, and STAT5 [55] when expressed in isolation in undifferentiated myeloid cells. It is interesting to note that ERK signalling is crucial for CREB phosphorylation at the MIEP and subsequent deposition of the activatory mark H3S10P on the MIEP upon differentiation-induced reactivation [22]. Consistent with this, and the ability of US28 to attenuate ERK signalling, infection of monocytes with ΔUS28 HCMV (which no longer suppresses the MIEP) is also associated with activated CREB and phosphorylated H3S10 on the MIEP. Furthermore, pharmacological inhibition of ERK in combination with NF-κB could prevent lytic replication of ΔUS28 HCMV in monocytes and, conversely, treatment of monocytes with small molecule inhibitors of US28 also results in a lytic infection rather than latency [55].

Attenuation of these cellular signalling pathways is reversed when US28-expressing cells are differentiated into macrophage-like cells using phorbol esters [55]. The implication then is that US28 helps to maintain latency via the attenuation of MIEP-activatory cascades but does not block signalling from these pathways during reactivation, and may even support their function during cellular differentiation. Indeed, in reporter systems, US28 represses the MIEP in undifferentiated THP-1 monocytes, but activates the MIEP in PMA-differentiated THP-1 derived macrophages [55].

Recent work has also shown that US28 decreases c-fos levels during latency. Binding to the AP-1 site within the MIEP by fos/jun dimers activates the MIEP, and so, in decreasing c-fos, US28 enacts MIEP suppression via an additional mechanism. As such, treatment of myeloid cells with a c-fos inhibitor reduced lytic gene expression when infecting with ΔUS28 HCMV [56].

Taken together, a key mechanism by which US28 supports latency in undifferentiated myeloid cells is to modulate multiple cellular signalling pathways, which alters the balance of activatory and repressive factors at the MIEP, the result of which is to promote a repressive chromatin structure at the MIEP and thus suppress IE gene expression. In contrast, this suppressive function of US28 does not occur in differentiated myeloid cells and so US28 does not promote a repressive chromatin structure at the MIEP during differentiation-induced reactivation.

## Targeting US28 represents a novel way to target the latent viral reservoir

Latent carriage of HCMV in myeloid progenitor cells provides a reservoir of reactivatable virus that cannot be cleared with current therapeutics. CMV reactivation events in immunocompromised patients can cause serious morbidity and mortality, particularly in the organ transplant setting. Clearing or reducing the latent reservoir in patients or donors may, therefore, be a way to reduce the burden of CMV-disease in transplant recipients.

Understanding the role of latency-associated gene products identifies viral genes that must be expressed during latency and thus represent potential strategies for targeting latently infected cells. Proof of principle for this came with the observation that UL138 reduces MRP1 on the cell surface of latently infected cells, and therefore the toxin vincristine was selectively taken up by latently infected cells [63].

US28 represents excellent potential for targeting the latent reservoir because (1) US28 is expressed on the cell surface during latency [48]; (2) G-protein coupled receptors are well-appreciated pharmacological targets; (3) US28 controls latency via the MIEP. Indeed, US28 on the surface of latently infected cells may be a target for antibody-dependent killing by autologous neutrophils, but HCMV evades this killing in part by downregulating neutrophil chemoattractants [64].

One strategy considered for US28-targetting was to link a high-affinity ligand for US28 with a toxin. Upon binding ligand, US28 is internalised, and thus would deliver the toxin into latently infected cell. By fusing part of the *Pseudomonas* Endotoxin A with CX3CL1 (also known as fractalkine), such a fusion toxin protein (F49A-FTP) was devised [65]. Since US28 has a higher affinity for CX3CL1 than the native receptor, F49A-FTP could selectively kill experimentally and naturally latent monocytes and reduce reactivation events in vitro [48]. Using F49A-FTP to flush out the latent reservoir in normothermic solid organs for transplant is currently under investigation.

A second strategy relies on the known function of US28 during latency. US28 suppresses the MIEP in myeloid cells, and the inverse agonist VUF2274 inhibits US28 function during latency, leading to reactivation [55]. Full reactivation of HCMV may not be desirable since HCMV encodes many immune evasins at later time points [66, 67] and would thus evade natural immune control by T and NK cells. Transient induction of IE gene expression might be considered preferable [68], since up to 5% of a seropositive individual’s CD8^+^ T cells are capable of recognising lytic IE antigen [69]. Furthermore, striking evidence from studies of murine cytomegalovirus indicates that IE antigen is recognised by cytotoxic CD8^+^ T cells [70, 71]. Interestingly, sporadic induction of IE gene expression is observed in vivo in the lungs of infected mice [72, 73], and these events have been linked to the T cell “memory inflation” phenomenon [74]. In vitro analyses of primary human cells have shown that HDAC inhibitors can transiently induce IE expression in latently infected monocytes, thus allowing autologous cytotoxic T cells from seropositive donors to recognise and kill these infected cells. The result is a reduction in latent carriage in this experimental model of latency [21]. Perhaps an US28 inhibitor that partially blocks US28-mediated suppression of the MIEP would transiently induce IE and allow recognition by cytotoxic T cells. This is currently under study in our own laboratory. Several groups are also developing alternative US28 inhibitors [75–77] which might provide a highly-selective US28-based shock and kill strategy in the transplant setting.

## Concluding remarks

A molecular understanding of human cytomegalovirus latency has revealed pathways and mechanisms which may be therapeutically targeted to reduce the burden of reactivation-associated CMV disease. Chromatin structure at the MIEP is crucial for the control of latency and reactivation, and targeting the cellular and viral factors, including US28, which regulate the MIEP directly or indirectly, is a strategy for potential reduction of the latent viral reservoir within patients.
